# Supplementary material for: Large magnetoelectric coupling in multiferroic oxide heterostructures assembled via epitaxial lift-off
Source: Nat Commun. 2020 Jun 24;11:3190. doi: 10.1038/s41467-020-16942-x (PMC7314756; doi:10.1038/s41467-020-16942-x)
Supplement: Supplementary file 1 — Supplementary Information [file 41467_2020_16942_MOESM1_ESM.pdf]

Supplementary Information
for
Large magnetoelectric coupling in multiferroic oxide heterostructures assembled via epitaxial lift-off

D. Pesquera[1,*], E. Khestanova[2], M. Ghidini[3,4,1], S. Zhang[1,5], A. P. Rooney[6], F. Maccherozzi[4], P. Riego[1,7,8], S. Farokhipoor[9], J. Kim[1], X. Moya[1], M. E. Vickers[1], N. A. Stelmashenko[1], S. J. Haigh[6], S. S. Dhesi[4] and N. D. Mathur[1,†]

[1]Department of Materials Science, University of Cambridge, Cambridge, CB3 0FS, UK
[2]ITMO University, Saint Petersburg 197101, Russia
[3]Department of Mathematics, Physics and Computer Science, University of Parma, 43124 Parma, Italy
[4]Diamond Light Source, Chilton, Didcot, Oxfordshire, OX11 0DE, UK
[5]College of Science, National University of Defense Technology, Changsha 410073, China
[6]School of Materials, University of Manchester, Manchester M13 9PL, UK
[7]CIC nanoGUNE, E-20018 Donostia-San Sebastian, Spain
[8]Department of Condensed Matter Physics, University of the Basque Country, UPV/EHU, E-48080 Bilbao, Spain
[9]Zernike Institute for Advanced Materials, University of Groningen, 9747 AG Groningen, The Netherlands

*dpesquera@cantab.net, †ndm12@cam.ac.uk

Supplementary Notes

1. **In-plane orientation of PMN-PT substrate and transferred LSMO film**
2. **The interfacial layer between transferred LSMO and platinized PMN-PT**
3. **Lattice parameters for the precursor LSMO/SRO//STO sample**
4. **Surface topography of transferred LSMO and platinized PMN-PT**
5. **Cracks in the LSMO film**
6. **Curie temperature enhancement in LSMO following strain release**
7. **XRD measurements of the transferred LSMO film**
8. **Electrically driven phase transition in PMN-PT**
9. **Creation of the A and B remanent states in LSMO:PMN-PT**
10. **Reproducibility of macroscopic magnetoelectric measurements**
11. **Influence of cracks on the magnetic domain structure**
12. **Simulation of magnetoelectric effects**

Experimental data in all Notes were obtained at room temperature, except Note 6.

Supplementary Note 1

In-plane orientation of PMN-PT substrate and transferred LSMO film

In-plane phi-scans around high-intensity reflections (Supplementary Figure 1) confirmed a 2-fold symmetry in our PMN-PT $(011)_{pc}$ substrates, and revealed 4-fold symmetry in our transferred LSMO $(001)_{pc}$ films. A comparison of phi-scans reveals that the high-symmetry axes ($[010] \parallel b$ in LSMO, $[01\bar{1}] \parallel y$ in PMN-PT) were misaligned by 5° (Samples A,B) or 20° (Sample C).

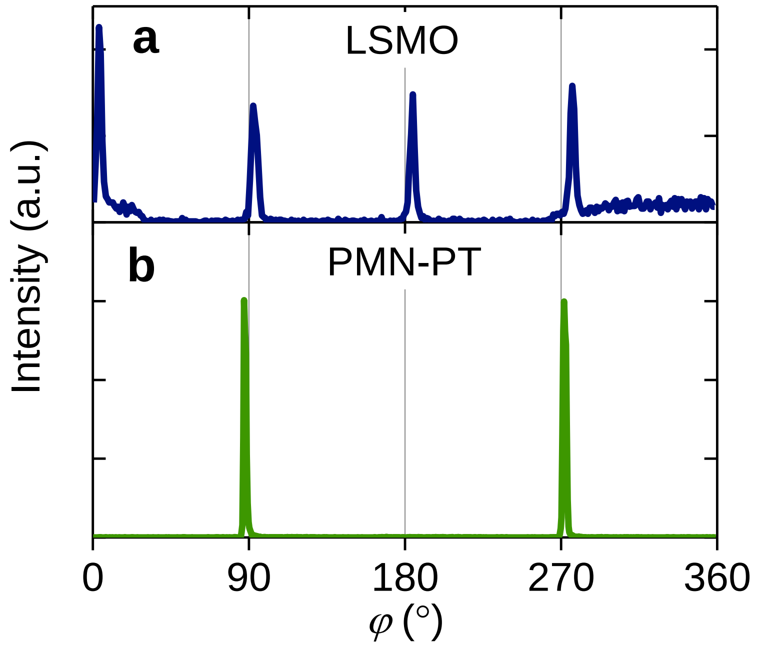

Supplementary Figure 1. In-plane phi-scans around high-intensity reflections in LSMO and PMN-PT. (a) Family of 011_{pc} reflections for LSMO. (b) Family of 013_{pc} reflections for PMN-PT. The misorientation between the crystal axes of two materials is 5°. Data for Sample A.

Supplementary Note 2

The interfacial layer between transferred LSMO and platinized PMN-PT

Cross-sectional scanning transmission electron microscopy (STEM) revealed the presence of a 10 nm-thick amorphous layer between the LSMO film and top electrode of the platinized PMN-PT substrate (Supplementary Figure 2a). This amorphous layer, which mediates the strain-mediated magnetoelectric effects that we observed, was found to be flat and uniform over several microns in our STEM lamella. The maximum thickness variation was 2 nm, and we may infer that contaminants did not self-assemble into large pockets[1].

Elemental analysis revealed that the amorphous layer contained Si, O and accumulation of C at both interfaces (Supplementary Figure 2b). We attribute these residuals to the partial degradation of the PDMS membrane during the $SrRuO_3$ etch (no residuals of Sr or Ru are apparent). Carbon may also be present near the Pt electrode due to adsorbed hydrocarbons, which typically become trapped between substrates and transferred two-dimensional crystals[2,3].

Electron energy loss spectroscopy (EELS) images obtained at the Mn L_3/L_2 edges (not shown) did not identify a change of Mn oxidation state in the LSMO film near its interface with the amorphous layer. This implies a weak van der Waals-like bonding rather than chemical reaction, as suggested for LSMO transferred to Si substrates[4].

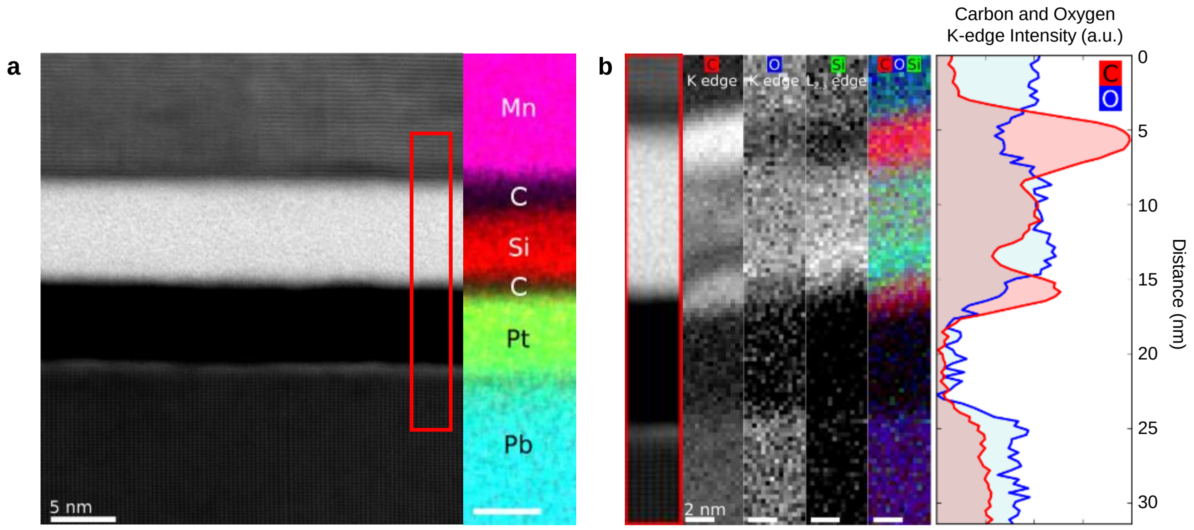

Supplementary Figure 2. Detail showing the amorphous layer between LSMO and the platinized PMN-PT substrate. (a) Bright-field cross-sectional STEM image for LSMO:PMN-PT, and energy dispersive x-ray (EDX) image of the adjacent region showing selected elements in each layer. Scale bars in (a) are 5 nm. (b) The region highlighted red in (a) is magnified at left. The other panels in (b) show EELS data for this area, namely images obtained at the as-specified carbon, oxygen and silicon edges (presented separately and together); and the K-edge intensity distribution for C and O (normalised counts do not indicate relative composition). Scale bars in (b) are 2 nm. All data for sample C.

Supplementary Note 3

Lattice parameters for the precursor LSMO/SRO//STO sample

A reciprocal space map around the asymmetric 103 STO reflection yields the in-plane lattice parameter *a* and its out-of-plane counterpart *c* for all three materials in the precursor sample (Supplementary Table 1). The in-plane lattice parameters for all three materials match well, indicating that the SRO and LSMO films both grew coherently on the STO substrate.

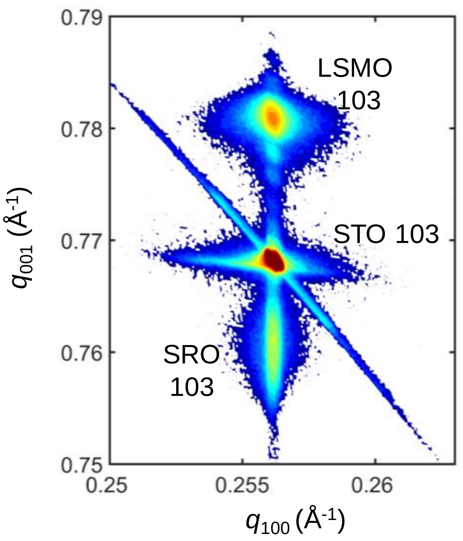

Supplementary Figure 3. XRD reciprocal space map around the STO 103 reflection. The in-plane lattice parameters for all three materials are similar. STO is cubic, SRO and LSMO are indexed as pseudocubic. Intensity scale runs from blue (low) to red (high).

	a (Å)	*c* (Å)
STO	3.906	3.904
SRO	3.905	3.944
LSMO	3.905	3.841

Supplementary Table 1. Lattice parameters for all three materials. Cubic (STO) and pseudocubic (SRO and LSMO) lattice parameters *a* and *c* are given for the in-plane and out-of-plane directions, respectively.

Supplementary Note 4

Surface topography of transferred LSMO and platinized PMN-PT

Atomic force microscopy images of the transferred LSMO film (Supplementary Figure 4a) and an exposed region of the platinized PMN-PT substrate (Supplementary Figure 4b) are both dominated by topographical features that arise from ferroelectric domains spanning roughly 1-5 μm.

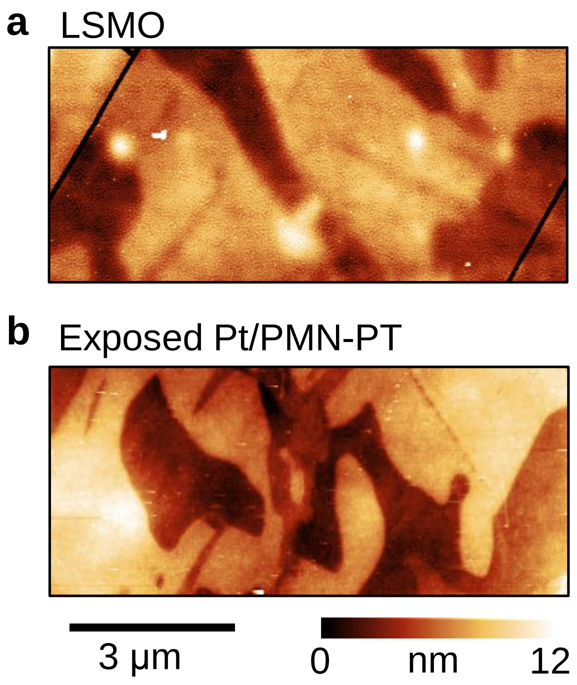

Supplementary Figure 4. Surface topography for LSMO:PMN-PT. Atomic force microscopy (AFM) images of (a) transferred LSMO and (b) the exposed Pt/PMN-PT. Data from sample B.

Supplementary Note 5

Cracks in the LSMO film

We observed randomly oriented cracks in the LSMO film after transfer to the flexible PDMS membrane (inset, Supplementary Figure 5). It therefore follows that we observed cracks in the LSMO film after it had been subsequently transferred to the platinized PMN-PT substrate (Supplementary Figure 5).

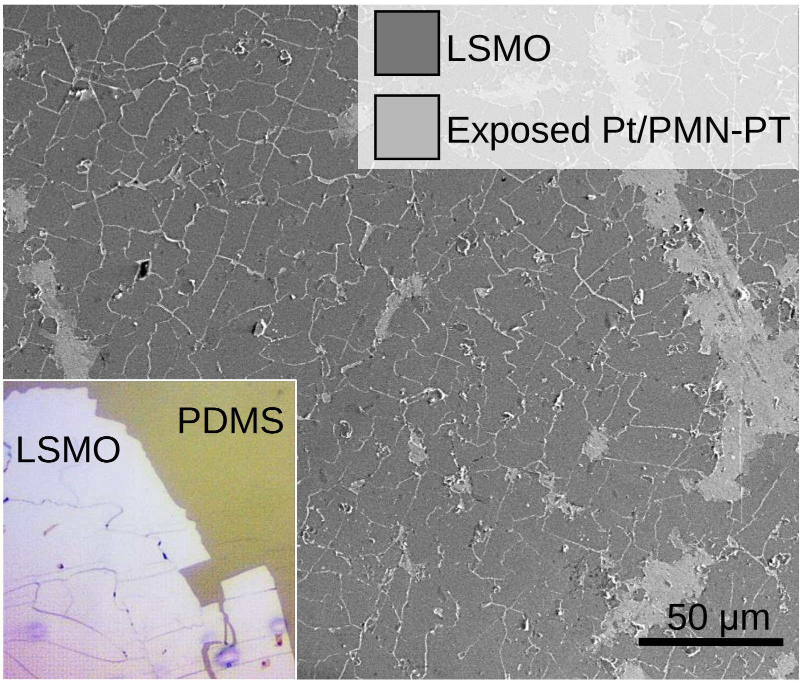

Supplementary Figure 5. Cracks in the LSMO film during and after transfer. Scanning electron microscopy (SEM) image of LSMO:PMN-PT showing the transferred LSMO film (dark) and exposed regions of the platinized PMN-PT substrate (bright). Inset: optical microscopy image of the same LSMO film on the PDMS membrane before transfer to the platinized PMN-PT (no scale recorded). Data for Sample C (main image) and precursor LSMO (inset).

Supplementary Note 6
Curie temperature enhancement in LSMO following strain release

The Curie temperature of LSMO was enhanced by the release of epitaxial growth strain, as determined after subsequent transfer to PMN-PT (Supplementary Figure 6).

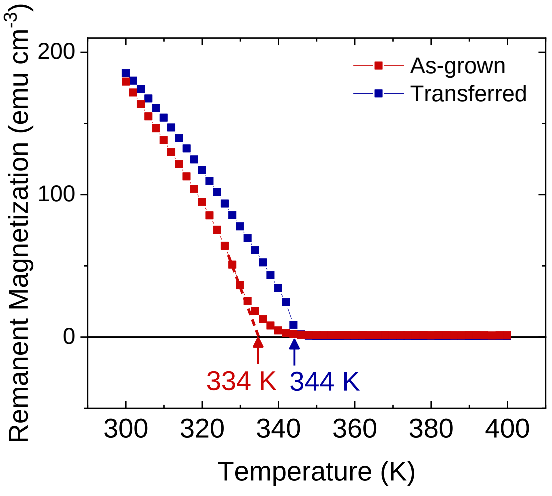

Supplementary Figure 6. Curie temperature enhancement in LSMO following strain release. Temperature-dependent remanent magnetization for LSMO in LSMO/SRO//STO after growth (red) and in LSMO:PMN-PT after transfer (blue). Data were obtained on warming in zero magnetic field in a SQUID magnetometer, after having applied a saturating in-plane field of 400 Oe along an arbitrary direction at room temperature. The samples used here are similar to those described elsewhere in this paper, permitting qualitative comparison only.

Supplementary Note 7

XRD measurements of the transferred LSMO film

Our transferred LSMO film displayed split x-ray reflections (Supplementary Figure 7a,b), permitting us to infer that it comprised twins of orthorhombic LSMO with different in-plane lattice parameters.

All four reflections that we show in Supplementary Figure 7 undergo small shifts on electrically switching between the remanent states that we label A and B. These shifts are only just resolved, which we infer to be a consequence of measuring small twins on a topography determined by the underlying ferroelectric domains.

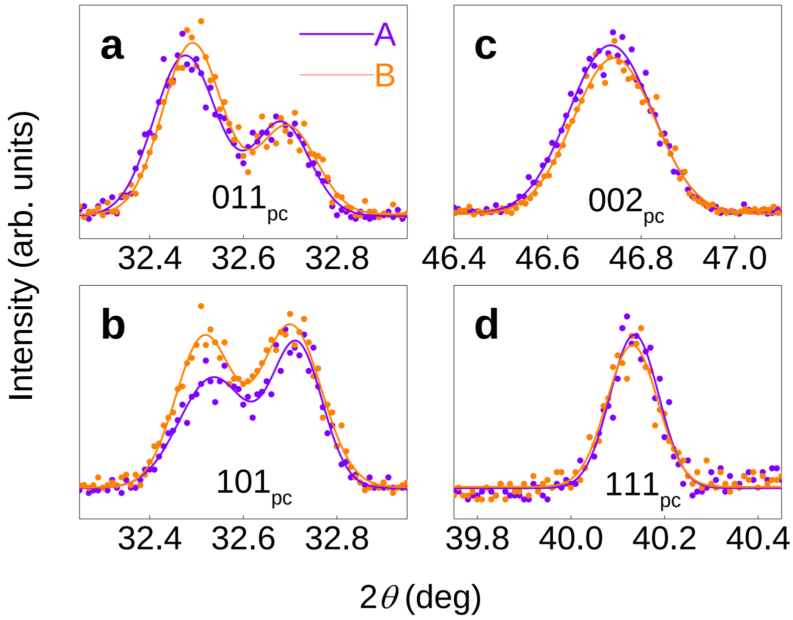

Supplementary Figure 7. Structure of the LSMO film at remanent states A and B in LSMO:PMN-PT. (a-d) X-ray diffraction 2θ-ω scans showing four LSMO reflections in remanent states A (purple) and B (orange). Lines are fits to data. Data for Sample A.

Supplementary Note 8
Electrically driven phase transition in PMN-PT

Here we show that electrically induced switching of electrical polarization (Supplementary Figure 8a) and macroscopic strain (Supplementary Figure 8b,c) are associated with the interconversion of orthorhombic (O) and rhombohedral (R) phases. We identify this interconversion from reciprocal space maps (Supplementary Figure 8d,e) for the $(222)_{pc}$ and $(031)_{pc}$ lattice planes (Supplementary Figure 8f), whose asymmetric x-ray reflections undergo complementary splitting (Supplementary Figure 8g,h) due to the presence of domains (Supplementary Figure 8i,j).

Specifically, our reciprocal space maps for the 222_{pc} (Supplementary Figure 8d) and 031_{pc} (Supplementary Figure 8e) reflections reveal that:

- There was essentially a single broad peak after thermal depolarization.
- On applying and removing -10 kV cm^{-1}, there was a single 222_{pc} reflection and a split 031_{pc} reflection, implying the formation of $O_{1,2}$ domains.
- On approaching the peak strain at 2.67 kV cm^{-1}, the single 222_{pc} reflection observed after poling was replaced by a split peak, and the split 031_{pc} reflection observed after poling was replaced by a single peak, implying the formation of $R_{1,2}$ domains. The concomitant formation of minority $R_{3,4}$ domains with in-plane polarizations[5] is evidenced by the creation and subsequent destruction of an additional 031_{pc} reflection with low intensity.
- At 3.33 kV cm^{-1}, the $O_{1,2}$ domains began to reappear.
- At higher fields, the switching current approached zero, the strain approached saturation, the $O_{1,2}$ domains were re-established and the R phase was eliminated.

The two strain states that we achieved at electrical remanence[6] (Fig. 2b in main paper) are therefore identified with the O and R phases in our major loop here as follows:

- The A state obtained after removing a saturating field is identified with the O phase observed here after poling.
- The B state obtained after reaching the coercive field on a major loop and then removing the applied field is identified with the R phase observed here near the coercive field.

Note that states A and B were obtained after positive poling (Fig. 2b in main paper), while the corresponding states identified here were obtained after negative poling (Supplementary Figure 8d,e), such that permitted directions of polarization (red arrows, Supplementary Figure 8i,j) are reversed.

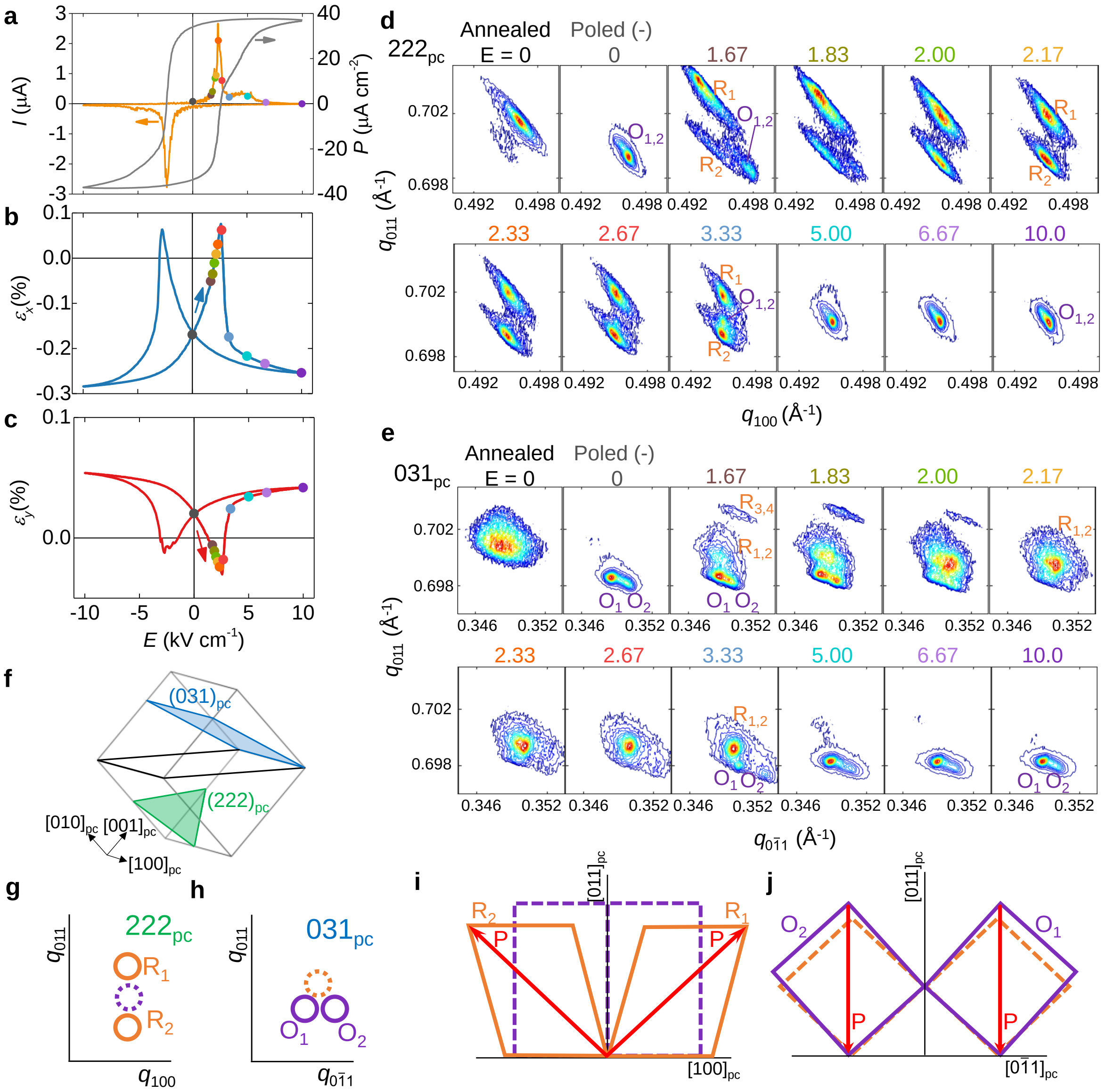

Supplementary Figure 8. Electrically driven phase transition in PMN-PT. (a-c) For major loops of electric field E, we show (a) polarization P (grey) deduced from switching current I (orange), and in-plane strains (b) ε_x and (c) ε_y. Coloured points identify coloured kV cm^{-1} values of E in (d,e), where we show reciprocal space maps around the (d) 222_{pc} and (e) 031_{pc} reflections obtained first at zero-field after a rejuvenating anneal in air at 150 °C, then at zero-field after negative poling, and then at increasing values of E. Intensity scale runs from purple (low) to red (high). Scattering vector $q = (2/\lambda)\sin(\theta)$ for Bragg angle θ. Data in (a-c) acquired simultaneously using PMN-PT from the same master as sample A. Data in (a-e) for Sample A. (f) Pseudocubic unit cell showing the $(011)_{pc}$ surface (two bold black lines) and the $(222)_{pc}$ and $(031)_{pc}$ lattice planes. (e-j) For the R-phase (orange) and O-phase (purple), schematics of the reciprocal space maps show the (g) 222_{pc} and (h) 031_{pc} reflections, which are split (solid outlines) or unsplit (dashed outlines) according to whether (i,j) the projections of the unit cell over the corresponding real-space planes present twinning (solid outlines) or not (dashed outlines). Red arrows in (i,j) denote permitted directions of polarization.

Supplementary Note 9

Creation of the A and B remanent states in LSMO:PMN-PT

The four-fold anisotropy in LSMO after transfer (Fig. 1e of the main paper, reproduced in Supplementary Figure 9a) was modified by 30 bipolar cycles that each comprised major or minor loops of the type used to measure macroscopic magnetoelectric effects (Fig. 3 of the main paper). The inclusion of minor loops permitted access to remanent states A and B, whose evolution is shown in Supplementary Figure 9b-d.

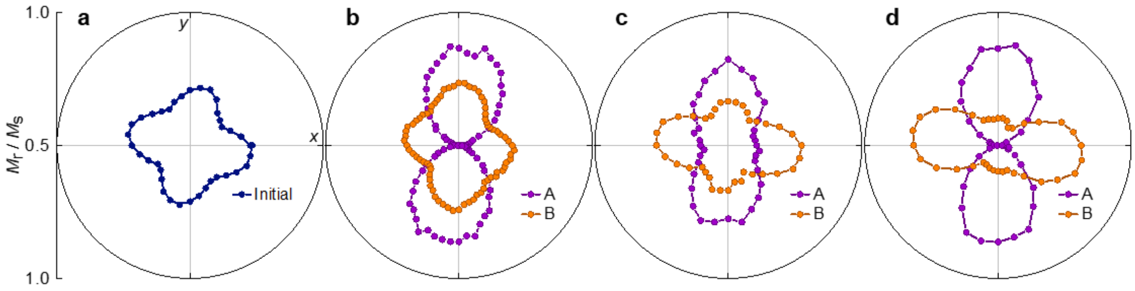

Supplementary Figure 9. Creation of the A and B remanent states in LSMO:PMN-PT. (a-d) Polar plots of loop squareness M_r/M_s based on data obtained (a) for the virgin state, and then after (b) 2, (c) 5 and (d) 30 bipolar cycles (that each comprised either a major loop accessing remanent state A, or a minor loop accessing remanent states A and B). Magnetic remanence is denoted M_r, saturation magnetization is denoted M_s. Data in (a) matches data in Fig. 1e of the main paper. Data in (d) matches data in Fig. 4c,d of the main paper.

Supplementary Note 10

Reproducibility of macroscopic magnetoelectric measurements

Major loops of $M_x(E)$ for sample A (Fig. 3 in the main paper) and two similar samples are similar (Supplementary Figure 10).

Supplementary Figure 10 also shows that magnetoelectric effects are completely suppressed by a saturating magnetic field. This confirms our expectation[7] that there are no strain-driven changes in the magnitude of the local magnetic moment, consistent with the magnetic rotations that we observed (Fig. 5 in the main paper).

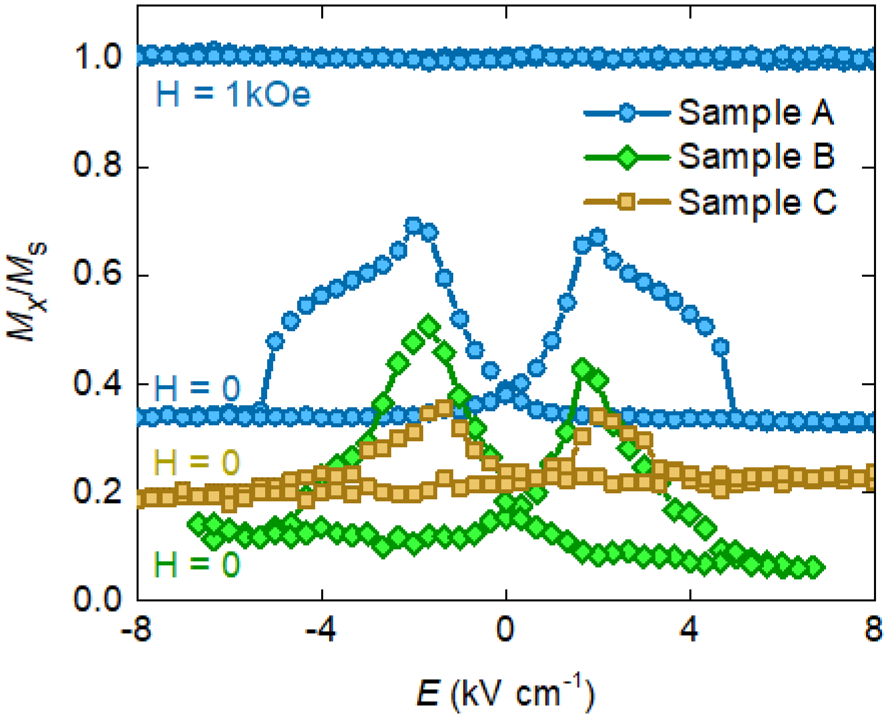

Supplementary Figure 10. Macroscopic magnetoelectric effects in LSMO:PMN-PT. In-plane magnetization component M_x normalized by saturation magnetization M_s versus electric field E. The lower three plots were measured for Samples A-C in zero magnetic field H, after applying and removing $H = 1$ kOe along x. The uppermost plot was measured for Sample A with $H = 1$ kOe along x.

Supplementary Note 11

Influence of cracks on the magnetic domain structure

In three regions of the LSMO:PMN-PT sample, cracks (left panels in Supplementary Figure 11) were found to coincide with a subset of magnetic domain perimeters (right panels in Supplementary Figure 11), suggesting that cracks are at least partially responsible for the coercivity enhancement in the transferred film.

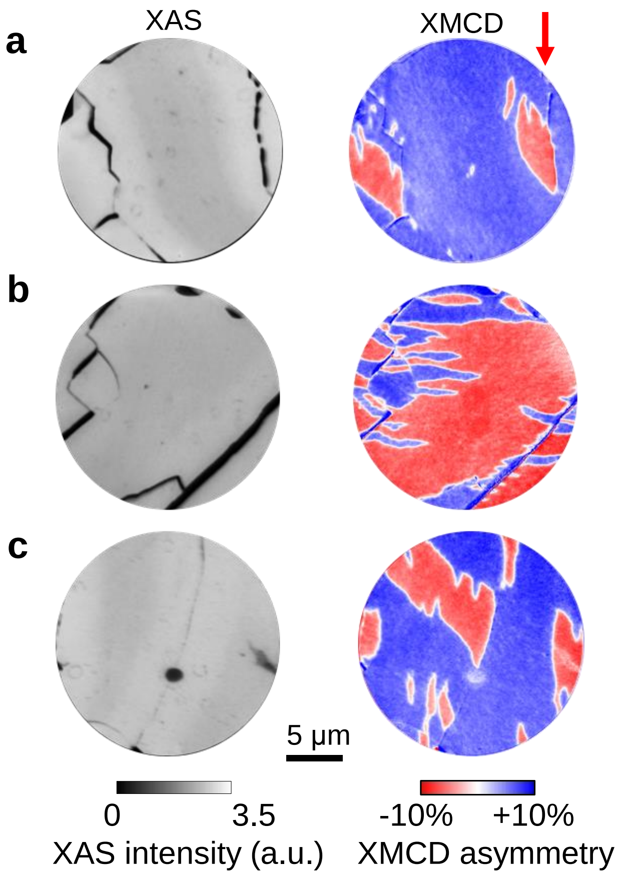

Supplementary Figure 11. Correlation between cracks and magnetic domains in LSMO:PMN-PT. Photoemission electron microscopy (PEEM) images for three different areas of the sample, with contrast from (left) x-ray absorption spectroscopy (XAS) and (right) x-ray magnetic circular dichroism (XMCD). Red arrow denotes the in-plane projection of the grazing-incidence x-ray beam. Data for Sample A.

Supplementary Note 12
Simulation of magnetoelectric effects

First, we will present a free energy density that describes the magnetoelectric effects in our transferred LSMO film, where the observed inhomogeneity is attributed to an invariant uniaxial stress anisotropy arising from the transfer. Then we will use this free energy density to simulate macroscopic and microscopic magnetoelectric effects, for comparison with our experimental observations.

Free energy density

The inhomogeneity that we observed in our XMCD-PEEM vector maps (Fig. 5 in the main paper) evidences different types of region in our transferred LSMO film. We will arbitrarily consider there to be nine types of region, and we will neglect exchange coupling between adjacent regions. The free energy density F for the LSMO film may then be written as:

$$F(E) = \sum_{i=1}^{9} F_i(\phi_i, E), \tag{1}$$

where F_i denotes the total free energy density for all distributed regions of the ith type, where the local magnetization in regions of the ith type adopts direction ϕ_i, where the magnitude of the local magnetization is equal to the saturation magnetization M_s = 425 emu cm^{-3} of the transferred LSMO film (blue data, Fig. 1d in the main paper), and where E denotes the electric field applied across the PMN-PT substrate. The function $F_i(\phi_i,E)$ is given by:

$$F_i(\phi_i, E) = -K_c \sin^2(\phi_i - \alpha)\cos^2(\phi_i - \alpha) - K_s(\varepsilon_{\mathrm{eff}}(E))\cos^2(\phi_i - \beta) - K_u \cos^2(\phi_i - \varphi_i), \tag{2}$$

where each term on the right is described below. Positive angles (ϕ_i, α, β and φ_i) imply anticlockwise rotations with respect to the x direction in PMN-PT as viewed from the LSMO film.

Magnetocrystalline anisotropy

The term $-K_c \sin^2(\phi_i - \alpha)\cos^2(\phi_i - \alpha)$ is common to all nine types of region in the LSMO film, and describes the four-fold magnetocrystalline anisotropy (ref. 45 in the main paper), for which $\alpha = 40°$ is one of the hard directions (Fig. 1e in the main paper). The anisotropy constant $K_c = H_a M_s/2$= 3.5 kJ m^{-3} was estimated from the values of anisotropy field H_a = 165 Oe and

saturation magnetization M_s = 425 emu cm^{-3} that we measured for the transferred LSMO film (blue data, Fig. 1d in the main paper). Supplementary Figure 12a shows a polar plot of the magnetocrystalline anisotropy density for the LSMO film.

Piezostress anisotropy

The term $-K_s(\varepsilon_{eff}(E))\cos^2(\phi_i - \beta)$ is common to all nine types of region in the LSMO film, and describes the uniaxial stress anisotropy due to piezoelectric strain from the substrate. For this term, β = 0° is the hard direction for the negative value of anisotropy constant $K_s(\varepsilon_{eff}(E))$ in state A, and β = 90° is the hard direction for the positive value of anisotropy constant $K_s(\varepsilon_{eff}(E))$ in state B. The anisotropy constant $K_s(\varepsilon_{eff}(E))=\frac{3}{2}\lambda E_Y\varepsilon_{eff}(E)$ changes sign due to electrically driven changes in the effective strain[8] $\varepsilon_{eff}(E) = \eta[\varepsilon_x(E) - \varepsilon_y(E)]/(1 + v)$ experienced by the LSMO film, where:

- $\varepsilon_x(E)$ and $\varepsilon_y(E)$ are the electric-field dependent strains transmitted by the PMN-PT substrate (Fig. 2a in the main paper);
- strain-transfer coefficient η = 40% was found to be reasonable by comparing our results for $M_x(E)$ and $M_y(E)$ (Supplementary Figure 13) with our macroscopic measurements (Fig. 3 in the main paper);
- the LSMO Poisson's ratio v = 0.33 was calculated from the strain in our epitaxial LSMO film prior to transfer (the XRD data in Fig. 1c in the main paper implies an out-of-plane pseudocubic lattice parameter of 3.841 Å, the $SrTiO_3$ substrate implies an in-plane pseudocubic lattice parameter of 3.905 Å, and the bulk lattice parameter of LSMO was taken to be[9] 3.873 Å);
- we assume $\varepsilon_z = 0$;
- LSMO magnetostriction[10] $\lambda = 3 \times 10^{-5}$;
- LSMO Young's modulus[11] E_Y = 100 GPa.

Supplementary Figure 12b shows a polar plot of the electrically controlled stress anisotropy density for the LSMO film.

Constant stress anisotropy

The term $-K_u\cos^2(\phi_i - \varphi_i)$ describes a uniaxial stress anisotropy, which we assume to arise in the ith type of region due to stress arising from the transfer of the LSMO film (we set $K_u = K_c$). The nine types of region are characterized by nine equally separated directions $\varphi_i = \frac{\pi i}{9}$. Each

of these directions describes an easy axis for $-K_\mathrm{u}\cos^2(\phi_i - \varphi_i)$, and collectively the nine directions average to zero (consistent with our macroscopic measurements of strain and magnetic anisotropy). Supplementary Figure 12c shows polar plots of the constant stress anisotropy for each type of region. The free energy density $F_i(\phi_i,E)$ for each type of region is shown in Supplementary Figure 12d for state A, and in Supplementary Figure 12e for state B.

The free energy density $F(E)$ for the LSMO film displays a two-fold anisotropy (Fig. 12f), with the easy axis along y (state A) or x (state B), as observed experimentally (Fig. 4 in the main paper).

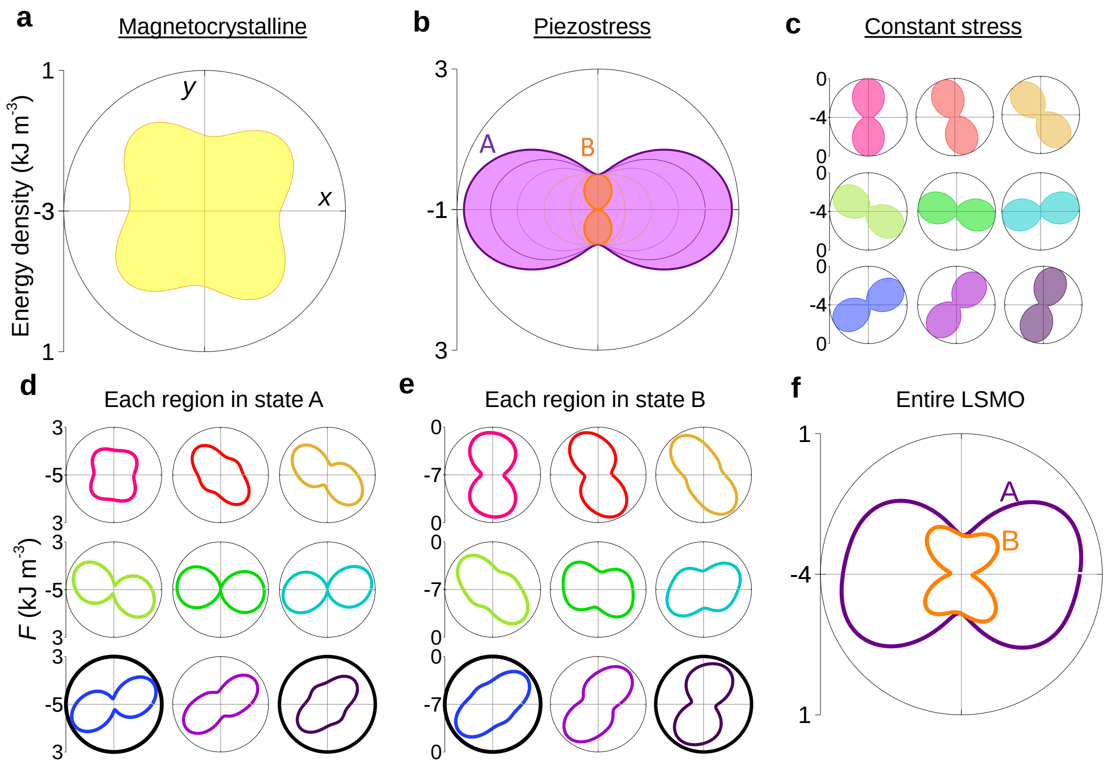

Supplementary Figure 12. Polar plots of energy density. (a) Polar plot of the magnetocrystalline anisotropy density, which is common to all nine types of region in the LSMO film. (b) Polar plot of the electrically controlled stress anisotropy density, which is common to all nine types of region in the LSMO film, and differs for states A and B. (c) Polar plots of the constant stress anisotropy density for the nine types of region in our LSMO film that encode the observed inhomogeneity. (d,e) Polar plots of the free energy density $F_i(\phi_i,E)$ for all nine regions when the sample adopts (d) state A and (e) state B (data obtained by summing the plots in (a-c), the four plots with bold outlines are shown in the main paper as Fig. 5g,h). (f) Polar plot of the free energy density $F(E)$ for the entire LSMO film, when the sample adopts states A and B (data obtained by summing the plots in (d) and (e), respectively).

Simulation of macroscopic magnetoelectric effects

The free energy density $F(E)$ for the LSMO film was used to simulate plots of $M_x(E)$ and $M_y(E)$ at magnetic remanence, for comparison with our macroscopic magnetoelectric measurements (Fig. 3 in the main paper).

To account for the initial application and removal of the saturating magnetic field along the measurement axis, we added the magnetostatic energy density $-M_sH\cos(\phi_i - \gamma)$ to the free energy density $F_i(\phi_i,E)$ for each type of region. Here, H = 1000 Oe denotes the magnitude of the saturating field that we used in our experiments, γ denotes the measurement direction ($\gamma = 0°$ for x and $\gamma = 90°$ for y), and M_s = 425 emu cm^{-3} (see earlier).

After thus obtaining the nine values of ϕ_i that describe the state of the film at magnetic remanence, we used the following three steps to identify $M_x(E)$ and $M_y(E)$ (Supplementary Figure 13). First, we used our experimentally obtained plots of $\varepsilon_x(E)$ and $\varepsilon_y(E)$ (Fig. 2a in the main paper) to obtain $\varepsilon_{eff}(E)$. Second, we used $\varepsilon_{eff}(E)$ to minimize $F_i(\phi_i,E)$ for each type of region, and thus obtain the nine magnetization directions ϕ_i for every tenth experimental value of E. Third, we summed the projections of the nine magnetization vectors along x to obtain $M_x(E)$, and along y to obtain $M_y(E)$.

Our plots of $M_x(E)$ and $M_y(E)$ (Supplementary Figure 11) are similar to our experimental results (Fig. 3 in the main paper). The constant stress anisotropy term is responsible for rendering the peaks in $M_x(E)$ and the troughs in $M_y(E)$ as broad as their experimental counterparts. Moreover, the constant stress anisotropy term renders the plots of $M_x(E)$ and $M_y(E)$ repeatable on further electric-field cycling.

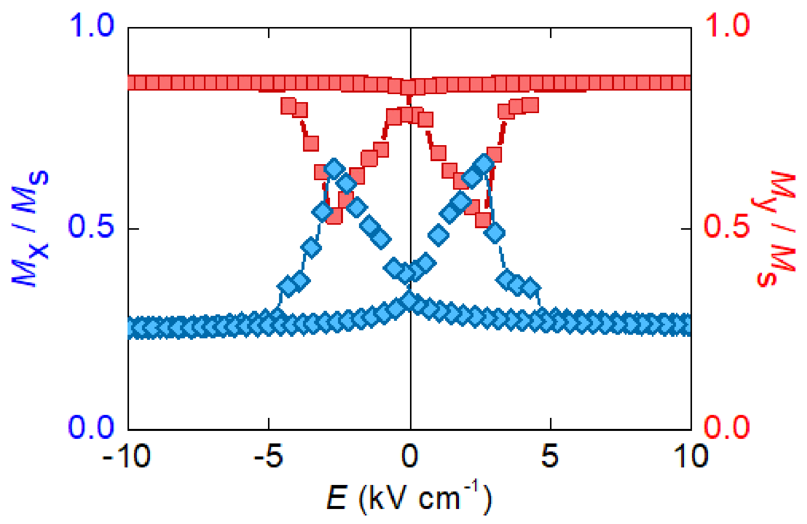

Supplementary Figure 13. Calculated magnetoelectric effects. Predicted plots of $M_x(E)$ and $M_y(E)$, normalized by saturation magnetization M_s.

Simulation of microscopic magnetoelectric effects

The free energy density $F(E)$ for the LSMO film was used to simulate electrically driven magnetic domain rotations in zero-magnetic field, for comparison with our corresponding microscopic observations based on XMCD-PEEM vector maps (Fig. 5a-f in the main paper).

To identify the demagnetized starting state at $E = 0$, we set the initial directions of magnetization in each type of region at random, with the constraint that all nine directions be equally spaced in angle. We then minimised $F_i(\phi_i,E)$ for each type of region, in order to obtain nine values of ϕ_i that no longer averaged exactly to zero.

After thus obtaining the nine values of ϕ_i that describe the demagnetized starting state at $E = 0$, we used the following three steps to identify the electrically driven angular changes $\Delta\phi_i$ between states A and B (Supplementary Table 2, Supplementary Figure 14). First, we used our experimentally obtained plots of $\varepsilon_x(E)$ and $\varepsilon_y(E)$ (Fig. 2a in the main paper) to obtain $\varepsilon_{\mathrm{eff}}(E)$. Second, at every tenth experimental value of E, we used $\varepsilon_{\mathrm{eff}}(E)$ to minimize $F_i(\phi_i,E)$ for each type of region and thus obtain nine magnetization directions ϕ_i. Third, we identified the nine values of ϕ_i for states A and B in the electrical cycle.

The resulting values of $\Delta\phi_i$ reproduce key features of our experimental observations because they are widely distributed in magnitude (from a few degrees up to 62°), and because some rotation angles calculated here (Regions of type 7, 9 and 3, Supplementary Table 2) are similar to those observed experimentally (Regions 1-3, Fig. 5f in the main paper).

**Region of type *i***	φ_i **(º)**	ϕ_i **in state A (º)**	ϕ_i **in state B (º)**	$\Delta\phi_i$ **(º)**	**Colour**
**1**	0	176	178	2	■
**2**	20	-111	-173	-62	■
**3**	40	-109	-161	-52	■
**4**	60	-102	-112	-10	■
**5**	80	-95	-99	-4	■
**6**	100	-88	-87	1	■
**7**	120	-81	-68	13	■
**8**	140	-74	-21	53	■
**9**	160	108	169	61	■

Supplementary Table 2. Calculated magnetization directions. For the nine types of region defined by the direction φ_i of the constant uniaxial stress anisotropy, we give values of ϕ_i for states A and B. These values and the corresponding changes $\Delta\phi_i$ are presented in Supplementary Figure 12 using the colour code defined here.

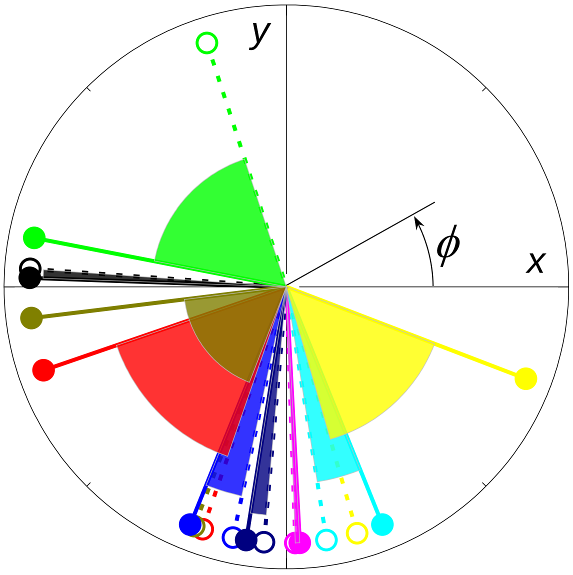

Supplementary Figure 14. Calculated magnetization directions. Visual presentation of the data in Supplementary Table 2. Values of ϕ_i in State A (empty circles) and State B (filled circles) differ by $\Delta\phi_i$ (shaded sectors).

Supplementary References

[1] Kretinin, A. V., Cao, Y., Tu, J. S., Yu, G. L., Jalil, R., Novoselov, K. S., Haigh, S. J., Gholinia, A., Mishchenko, A., Lozada, M., Georgiou, T., Woods, C. R., Withers, F., Blake, P., Eda, G., Wirsig, A., Hucho, C., Watanabe, K., Taniguchi, T., Geim, A. K. & Gorbachev, R. V. Electronic properties of graphene encapsulated with different two-dimensional atomic crystals. *Nano Lett.* **14,** 3270–3276 (2014).

[2] Haigh, S. J., Gholinia, A., Jalil, R., Romani, S., Britnell, L., Elias, D. C., Novoselov, K. S., Ponomarenko, L. A., Geim, A. K. & Gorbachev, R. Cross-sectional imaging of individual layers and buried interfaces of graphene-based heterostructures and superlattices. *Nat. Mater.* **11,** 764–767 (2012).

[3] Khestanova, E., Guinea, F., Fumagalli, L., Geim, A. K. & Grigorieva, I. V. Graphene bubbles on a substrate: Universal shape and van der Waals pressure. *Nat. Commun.* **7,** 12587 (2016).

[4] Lu, D., Baek, D. J., Hong, S. S., Kourkoutis, L. F., Hikita, Y. & Hwang, H. Y. Synthesis of freestanding single-crystal perovskite films and heterostructures by etching of sacrificial water-soluble layers. *Nat. Mater.* **15,** 1255–1260 (2016).

[5] Nan, T., Liu, M., Ren, W., Ye, Z. G. & Sun, N. X. Voltage control of metal-insulator transition and non-volatile ferroelastic switching of resistance in VO_x/PMN-PT heterostructures. *Sci. Rep.* **4,** 05931 (2014).

[6] Wang, Z., Wang, Y., Ge, W., Li, J. & Viehland, D. Volatile and nonvolatile magnetic easy-axis rotation in epitaxial ferromagnetic thin films on ferroelectric single crystal substrates. *Appl. Phys. Lett.* **103,** 132909 (2013).

[7] Moya, X., Hueso, L. E., Maccherozzi, F., Tovstolytkin, A. I., Podyalovskii, D. I., Ducati, C., Phillips, L. C., Ghidini, M., Hovorka, O., Berger, A., Vickers, M. E., Defay, E., Dhesi, S. S. & Mathur, N. D. Giant and reversible extrinsic magnetocaloric effects in $La_{0.7}Ca_{0.3}MnO_3$ films due to strain. *Nat. Mater.* **12,** 52–58 (2012).

[8] Lou, B. J., Liu, M., Reed, D., Ren, Y. & Sun, N. X. Giant Electric Field Tuning of Magnetism in Novel Multiferroic FeGaB/Lead Zinc Niobate - Lead Titanate (PZN-PT) Heterostructures. *Adv. Mater.* **21,** 4711–4715 (2009).

[9] Radaelli, P. G., Iannone, G., Marezio, M., Hwang, H. Y., Cheong, S. W., Jorgensen, J. D. & Argyriou, D. N. Structural effects on the magnetic and transport properties of perovskite $A_{1-x}A'_xMnO_3$ (x = 0.25, 0.30). *Phys. Rev. B* **56,** 8265–8276 (1997).

[10] Ziese, M., Semmelhack, H. C. & Busch, P. Sign reversal of the magnetic anisotropy in $La_{0.7}A_{0.3}MnO_3$ (A = Ca, Sr, Ba, □) films. *J. Magn. Magn. Mater.* **246,** 327–334 (2002).

[11] Darling, T. W., Migliori, A., Moshopoulou, E. G., Trugman, S. A., Neumeier, J. J., Sarrao, J. L., Bishop, A. R. & Thompson, J. D. Measurement of the elastic tensor of a single crystal of $La_{0.83}Sr_{0.17}MnO_3$ and its response to magnetic fields. *Phys. Rev. B* **57,** 5093–5097 (1998).
